# An Acetamide Derivative as a Camptothecin Sensitizer for Human Non-Small-Cell Lung Cancer Cells through Increased Oxidative Stress and JNK Activation

**DOI:** 10.1155/2016/9128102

**Published:** 2016-10-24

**Authors:** Han-Lin Chou, Yao Fong, Hsin-Hsien Lin, Eing Mei Tsai, Jeff Yi-Fu Chen, Wen-Tsan Chang, Chang-Yi Wu, Hui-Min David Wang, Hurng-Wern Huang, Chien-Chih Chiu

**Affiliations:** ^1^Department of Biotechnology, Kaohsiung Medical University, Kaohsiung 807, Taiwan; ^2^Institute of Biomedical Science, National Sun Yat-Sen University, Kaohsiung 804, Taiwan; ^3^Department of Thoracic Surgery, Chi-Mei Medical Center, Tainan 710, Taiwan; ^4^Research Center for Environment Medicine, Kaohsiung Medical University, Kaohsiung 807, Taiwan; ^5^Division of Hepatobiliary and Pancreatic Surgery, Department of Surgery, Kaohsiung Medical University Hospital, Kaohsiung 807, Taiwan; ^6^Department of Surgery, School of Medicine, College of Medicine, Kaohsiung Medical University, Kaohsiung 807, Taiwan; ^7^Department of Biological Sciences, National Sun Yat-Sen University, Kaohsiung 804, Taiwan; ^8^Graduate Institute of Biomedical Engineering, National Chung Hsing University, Taichung 402, Taiwan; ^9^The Graduate Institute of Medicine, Kaohsiung Medical University, Kaohsiung 807, Taiwan

## Abstract

In recent years, combination chemotherapy is a primary strategy for treating lung cancer; however, the issues of antagonism and side effects still limit its applications. The development of chemosensitizer aims to sensitize chemoresistant cancer cells to anticancer drugs and therefore improve the efficacy of chemotherapy. In this study, we examined whether N-[2-(morpholin-4-yl)phenyl]-2-{8-oxatricyclo[7.4.0.0,2,7]trideca-1(9),2(7),3,5,10,12-hexaen-4-yloxy}acetamide (NPOA), an acetamide derivative, sensitizes human non-small-cell lung cancer (NSCLC) H1299 cells towards camptothecin- (CPT-) induced apoptosis effects. Our results demonstrate that the combination of CPT and NPOA enhances anti-lung-cancer effect. The cytometer-based Annexin V/propidium iodide (PI) staining showed that CPT and NPOA cotreatment causes an increased population of apoptotic cells compared to CPT treatment alone. Moreover, Western blotting assay showed an enhancement of Bax expression and caspase cascade leading to cell death of H1299 cells. Besides, CPT and NPOA cotreatment-mediated disruption of mitochondrial membrane potential (MMP) in H1299 cells may function through increasing the activation of the stressed-associated c-Jun N-terminal kinase (JNK). These results showed that NPOA treatment sensitizes H1299 cells towards CPT-induced accumulation of cell cycle S phase and mitochondrial-mediated apoptosis through regulating endogenous ROS and JNK activation. Accordingly, NPOA could be a candidate chemosensitizer of CPT derivative agents such as irinotecan or topotecan in the future.

## 1. Introduction

Lung cancer is one of the common causes of death worldwide in both men and women [1]. Non-small-cell lung cancer (NSCLC) is one of the members of lung cancer types, and NSCLC accounts for about 80% of all lung cancers. Previous studies have shown that NSCLC often grows and spreads quickly [2]. Despite chemotherapy being one of the main therapeutic options for the treatment of NSCLC patients [3], the acquired chemoresistance often causes poor prognosis and recurrence in advanced NSCLC patients [4]. Therefore, improved efficacy and reduced cross-resistance of chemotherapeutic agents for NSCLC patients are urgent and are still under development [5].

Combination chemotherapies, a combination of two or more anticancer drugs, are aimed to enhance the efficacy of monochemotherapy [6]. However, the side effects of combination chemotherapies, such as neutropenia, febrile neutropenia, and sepsis, still cannot be avoidable completely during treatment, causing reduced efficacy of NSCLC treatments [7]. CPT, a quinoline alkaloid, is isolated from the Chinese ornamental tree* Camptotheca acuminata.* CPT and its derivatives are utilized to inhibit the proliferation of cancer cells, including lung cancer [8]. Two clinically used CPT derivatives irinotecan (CPT-11) and topotecan are widely used to inhibit the growth of lung cancer in combination with cisplatin, paclitaxel [9], and bevacizumab [10]. However, combination chemotherapy may cause antagonism and result in an attenuated efficacy of anticancer drugs [11, 12].

Chemotherapy sensitizers or chemosensitizers are used for enhancing the efficacy of anticancer drugs [13]. For example, PZ-39 (N-(4-chlorophenyl)-2-[(6-{[4,6-di(4-morpholinyl)-1,3,5-triazin-2-yl]amino}-1,3-benzothiazol-2-yl)sulfanyl]acetamide), an acetamide-containing compound, has been reported to significantly enhance the anti-breast-cancer activity effect of mitoxantrone, an anthracenedione [14]. Furthermore, amiloride, an acetamide-containing compound, enhances erlotinib-induced apoptosis of human pancreatic cancer cells [15]. Accordingly, the development of chemotherapy sensitizer could reduce antagonism and sensitize cancer cells to chemotherapeutic agents.

Acetamide-containing compounds are reported to exert various bioactivities, including anti-inflammatory, antibacterial, and antifungal activity [16–18]. For instance, 40006, an acetamide derivative, has been shown to inhibit inflammation of murine macrophage J774A.1 cells through reducing endogenous ROS [19]. Furthermore, acetamide derivatives have also been reported to exert anticancer activity [19, 20].* N*-Butyl-2-(2-fluorophenyl) acetamide (SCK6), an acetamide compound, has been reported to inhibit proliferation and to induce apoptosis of human lung squamous carcinoma CH27 cell via G_1_ cell cycle arrest [21]. Besides, combinations with acetamide derivatives and other chemotherapeutic agents have been reported to treat various cancer cells. For instance, the combination of acetamide derivatives N-(2-hydroxyphenyl) acetamide (NA-2) and Temozolomide (TMZ) synergistically induces apoptosis of human glioblastoma U87 cells [22], suggesting that acetamide derivatives could be a chemosensitizer for enhancing other chemotherapy agent-induced anticancer effects.

To overcome the limitations mentioned above of CPT-based treatment against NSCLC, we preliminarily examined a panel of acetamide derivatives and screened out a candidate N-[2-(morpholin-4-yl)phenyl]-2-{8-oxatricyclo[7.4.0.0,2,7]trideca-1(9),2(7),3,5,10,12-hexaen-4-yloxy}acetamide (NPOA). In this study, the synergistic effect and the mechanism of NPOA-mediated synergistic anti-NSCLC effect by CPT-based treatment were investigated. Furthermore, NPOA-mediated regulation of endogenous reactive oxygen species (ROS), the disruption of mitochondrial membrane potential (MMP), and the role of MAPK JNK in CPT-induced apoptosis of NSCLC cells were also discussed.

## 2. Materials and Methods

### 2.1. Preparation of Compounds

N-[2-(morpholin-4-yl)phenyl]-2-{8-oxatricyclo[7.4.0.0,2,7]trideca-1(9),2(7),3,5,10,12-hexaen-4-yloxy}acetamide (NPOA) was purchased from the chemical supplier Enamine™ (Kiev, Ukraine). CPT was purchased from Sigma-Aldrich (St. Louis, MO, USA). Both CPT and NPOA were dissolved in DMSO (below 0.01%) immediately before assays.

### 2.2. Reagents and Antibodies

DMEM with high glucose, F-12 Nutrient Mixture, trypan blue, dimethyl sulphoxide (DMSO), fetal bovine serum (FBS), antibiotics penicillin G, and streptomycin were purchased from Gibco BRL (Gaithersburg, MD, USA). Ribonuclease A (RNase A) was purchased from Sigma-Aldrich. Antibodies against phospho-JNK (Thr^183^/Tyr^185^) (#9101), total-JNK (#9102), cleaved caspase 9 (#7237), and cleaved caspase 3 (#9961) were purchased from Cell Signaling Technology (San Jose, CA, USA). Antibody against Bax (GTX61026) was obtained from Genetex (Alton Parkway, Irvine, CA, USA). Antibody against glyceraldehyde 3-phosphate dehydrogenase (GAPDH) was purchased from Santa Cruz Biotechnology Co. (Santa Cruz, CA, USA). Both anti-mouse and anti-rabbit IgG secondary antibodies conjugated to peroxidase were purchased from Leadgene Biomedical (#20102 and #20202, resp., Tainan, Taiwan). Annexin V-Fluorescein isothiocyanate (FITC) staining kit was purchased from Strong Biotech Co. (Taipei, Taiwan).

### 2.3. Cell Culture

Non-small-cell lung cancer cell lines A549 and H1299 were obtained from American Type Culture Collection (ATCC, Manassas, VA, USA). Cells were maintained in the medium DMEM: F-12 (ratio 3 : 2) and supplemented with 2 mM glutamine, 8% FBS, and the antibiotics 100 *μ*g/mL streptomycin and 100 units/mL penicillin at 37°C in a humidified condition of 5% CO_2_. Cells were checked using a PCR-based assay described previously for confirming no contamination of mycoplasma [23].

### 2.4. Proliferative Inhibition Assay

The proliferation rate was determined by a trypan blue exclusion-based assay. Briefly, 3 × 10^4^ NSCLC H1299 cells were seeded and treated with vehicle or the indicated concentrations of CPT and NPOA alone or cotreatment for 24 h and 48 h, respectively. After incubation, cells were stained with 0.2% trypan blue and counted by Countess™, an automatic cell counter (Invitrogen, Carlsbad, CA, USA).

### 2.5. Analysis of Drug Synergism

The drug synergism was analyzed according to the previous work [24]. Briefly, H1299 cells were treated with serial dilutions (from 0.125 to 1 *μ*M) of CPT combining with serial dilutions of NPOA (from 10 to 40 *μ*M). The values of combination index (CI) were analyzed using the software CalcuSyn (Biosoft, Cambridge, UK), a computer program based on the method of Chou and Talalay [25, 26]. The 95% confidence intervals for the dose-response values were used for determining the data. Synergism (CI < 1), additivity (CI = 1), or antagonism (CI > 1) presents the effect of drug combinations, respectively.

### 2.6. Apoptosis Assessment

Annexin V/PI dual staining was performed for detecting the externalization of phosphatidylserine (PS) from the cellular plasma membrane, a hallmark of apoptosis. Briefly, 1 × 10^5^ cells were seeded onto a 6-well plate and treated with CPT and NPOA alone or cotreatment for 24 h and 48 h, respectively. Subsequently, cells were harvested and stained with Annexin V/PI, and cells were analyzed by a flow cytometer Muse™ (Cell Analyzer, Merck Millipore, Billerica, MA, USA).

### 2.7. Oxidative Stress

The changes of intracellular redox state were determined by superoxide (O_2_^−^) sensitive fluorescent dye dihydroethidium (DHE) (Merck, Darmstadt, Germany). In brief, 1 × 10^5^ cells were seeded onto a 6-well plate and treated with CPT and NPOA alone or cotreatment for 6 h, respectively. Subsequently, cells were stained with 1 *μ*M DHE for 30 min in darkness. After incubation, the cells were analyzed by a flow cytometer Muse™ (Cell Analyzer, Merck Millipore).

### 2.8. Assessment of Mitochondrial Membrane Potential (ΔΨ*m*)

The changes of mitochondrial membrane potential (ΔΨ*m*) during cell apoptosis was determined by a cationic dye 5,5′,6,6′-tetrachloro-1,1′,3,3′-tetraethylbenzimi-dazol-carbocyanine iodide (JC-1) staining assay according to a previous study of Hsu et al.'s work with minor modifications [27]. The green fluorescence intensity of JC-1 is inversely proportional to the level of MMP (ΔΨ*m*). Briefly, H1299 cells (1 × 10^5^ cells/well) were seeded in a 6-well plate and treated with 0.5 *μ*M CPT alone and 10 *μ*M NPOA alone or their combination for 24 h, respectively. After incubation, cells were harvested and stained with 1 *μ*M of the JC-1 solution in serum-free medium and incubated at 37°C in the dark for 30 minutes. Afterward, cells were washed and detected by a flow cytometer Muse Cell Analyzer, Merck Millipore.

### 2.9. Western Blotting Assay

Western blotting assay was described previously with minor modifications [28]. In brief, 20 *μ*g protein was resolved by 10% SDS-polyacrylamide gel electrophoresis (SDS-PAGE) and then electrotransferred to a polyvinylidene fluoride (PVDF) membrane. The PVDF membrane was blocked with 5% nonfat milk in PBS-T buffer (PBS containing 0.1% Tween-20) and incubated with the primary and its corresponding secondary antibodies against specific proteins. The signals of specific protein were detected by a chemiluminescence-based WesternBright™ ECL detection solution (Advansta, Menlo Park, CA, USA).

### 2.10. Statistical Analysis

Differences between CPT and NPOA cotreatment and CPT-treated alone were analyzed in at least triplicated experiments. The difference was analyzed by one-way analysis of variance (ANOVA). ^*∗*^ *p* < 0.05 was considered statistically significant.

## 3. Results

### 3.1. CPT and NPOA Cotreatment Synergistically Enhances the Antiproliferation of H1299 Cells

**Figure 1:**
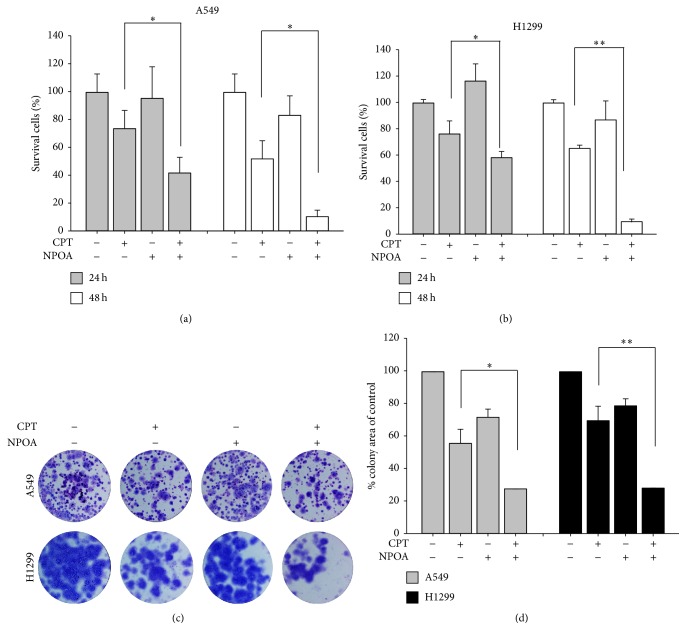
CPT and NPOA cotreatment inhibits cell proliferation of two NSCLC cells. The two NSCLC cell lines, A549 and H1299, were incubated with 0.5 *μ*M CPT and 10 *μ*M NPOA alone or in combination, respectively. (a and b) The rates of two NSCLC cell survival lines were determined using trypan blue exclusion assay. The quantification analysis of survival cells. (c) CPT and NPOA cotreatment inhibited the colony formation of two NSCLC cells for 11 days. Afterward, the cells were fixed in 4% paraformaldehyde and Giemsa stained. (d) The quantificative analysis of the colony area. Data are presented as means ± SD (^*∗*^ *p* < 0.05; ^*∗∗*^ *p* < 0.001).

To determine whether NPOA synergistically enhances CPT-induced antiproliferation of NSCLC cells, the multidrug effect analysis of Chou-Talalay method was used for analyzing the synergism of CPT and NPOA combination. The calculated 50% lethal concentration (LC_50_) of CPT for reducing cell viability is 0.5 *μ*M in H1299 cells. Besides, we found the treatment of NPOA was noncytotoxic in H1299 cells, whereas it synergistically enhanced CPT-induced cytotoxicity of H1299 cells (Supplementary Table  1 in Supplementary Material available online at http://dx.doi.org/10.1155/2016/9128102). Therefore, we next confirmed the synergism of CPT and NPOA combination on antiproliferation of NSCLC cell lines H1299 and A549 by trypan blue exclusion assay. As shown in Figures 1(a) and 1(b), the treatment of two compounds alone slightly inhibited the viability of two NSCLC cells for 24 h and 48 h. On the contrary, CPT and NPOA cotreatment markedly inhibited the viability of two NSCLC cells compared to both CPT and NPOA treatments alone, especially at 48 h treatment (^*∗∗*^ *p* < 0.001). Moreover, we performed colony formation assay to confirm the markedly inhibited cell proliferation of two NSCLC cells after CPT and NPOA cotreatment (Figures 1(c) and 1(d)).

### 3.2. NPOA Sensitizes NSCLC Cells towards CPT-Induced Mitochondrial-Mediated Apoptosis

**Figure 2:**
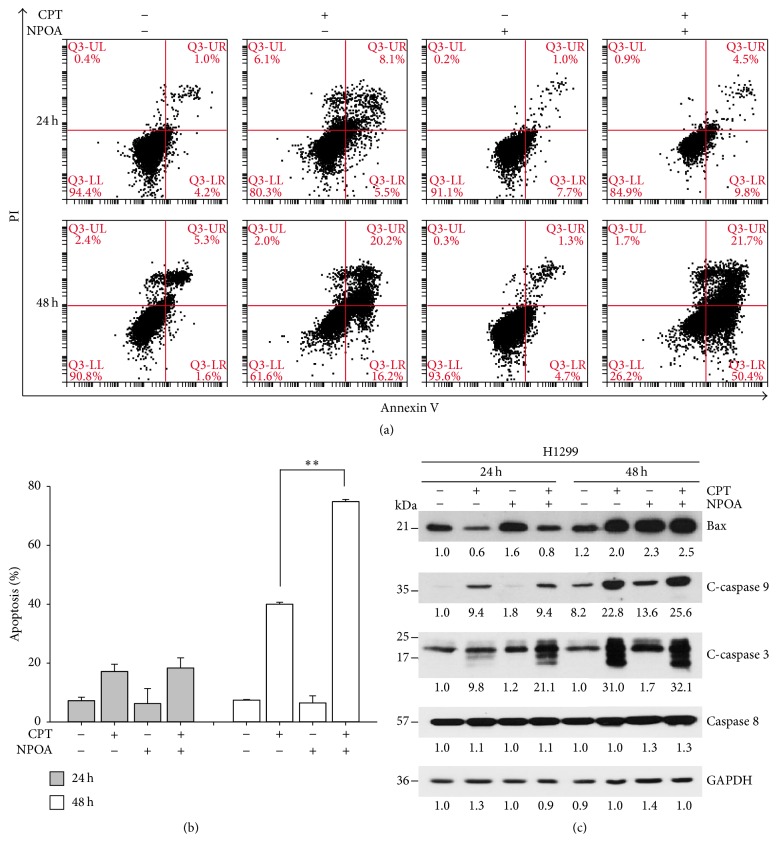
CPT and NPOA cotreatment induced apoptosis of H1299 cells. The cells were treated with 0.5 *μ*M CPT alone, 10 *μ*M NPOA alone, or their combination for 24 h and 48 h, respectively. (a) The cells were double-stained with Annexin V and PI and analyzed by flow cytometer-based detection assay. (b) The quantitative analysis of (a). Data are presented as means ± SD (^*∗∗*^ *p* < 0.001). (c) The results of Western blot assay showed the changes of mitochondrial apoptotic Bax protein, cleaved caspase 9 and cleaved caspase 3, and full-length caspase 8. Abbreviations: C-caspase 9 indicates cleaved caspase 9 and C-caspase 3 indicates cleaved caspase 3. GAPDH as an internal control for equal loading.

To determine whether combining CPT and NPOA inhibited cell growth by inducing apoptosis, the flow cytometer-based detection assay was determined by Annexin V/PI dual staining. In this assay, the percentages of Annexin V-positive/PI-negative were presented as early apoptosis, and the percentages of Annexin V-positive/PI-positive were presented as late apoptosis. The H1299 cells were incubated with indicated concentration of 0.5 *μ*M CPT alone, 10 *μ*M NPOA alone, or their combination for 24 h and 48 h, respectively. As shown in Figures 2(a) and 2(b), CPT treatment alone slightly induced apoptosis of H1299 cells at 24 h and 48 h, whereas the treatment of NPOA alone did not. However, CPT and NPOA cotreatment significantly increased the percentage of apoptotic cells in H1299 cells after 48 h treatment. In addition, the NPOA markedly enhanced CPT-induced apoptosis through regulating Bax and cleaved caspase 9 and caspase 3 (Figure 2(c)). These results showed that the NPOA synergistically enhanced CPT-induced apoptosis of H1299 cells.

### 3.3. CPT and NPOA Cotreatment Induces the Disruption of Membrane Potential in H1299 Cells

**Figure 3:**
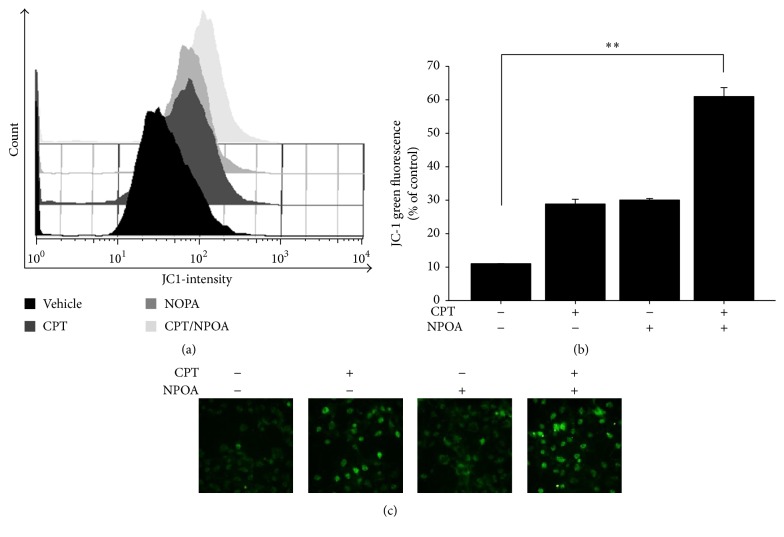
NPOA enhanced CPT-induced apoptosis through mitochondrial pathway. H1299 cells were treated with CPT alone and NPOA alone or their combination for 24 h, respectively. Afterward, H1299 cells were analyzed using flow cytometer- and fluorescence microscopy-based JC-1 assays, respectively. (a) The changes of mitochondrial membrane potential were determined by flow cytometer-based JC-1 staining assay. (b) The quantification analysis of (a). Data are presented as means ± SD (^*∗∗*^ *p* < 0.001). (c) The green fluorescence of JC-1 indicates the decrease of mitochondrial membrane potential, a hallmark of apoptosis at the early stage. Magnification 200x.

To determine whether CPT and NPOA cotreatment-induced apoptosis of NSCLC cells was through the modulation of mitochondria-mediated apoptosis pathway, JC-1, a cyanine dye, was used to detect the depolarization of mitochondrial membrane potential (MMP), a hallmark of mitochondrial-mediated apoptosis [29]. The H1299 cells were cultured with indicated concentration of 0.5 *μ*M CPT alone and 10 *μ*M NPOA alone or their combination for 24 h. As depicted in Figures 3(a) and 3(b), NPOA enhanced CPT-induced depolarization of MMP in H1299 cells. Moreover, the JC-1 fluorescence-based imaging assays also confirmed the synergism of NPOA on CPT-induced mitochondrial membrane depolarization of H1299 cells (Figure 3(c)). These results showed that a dramatical loss of MMP (ΔΨ*m*) was induced by CPT and NPOA cotreatment in H1299 cells.

### 3.4. NPOA Enhances CPT-Induced Endogenous ROS Production of H1299 Cells

**Figure 4:**
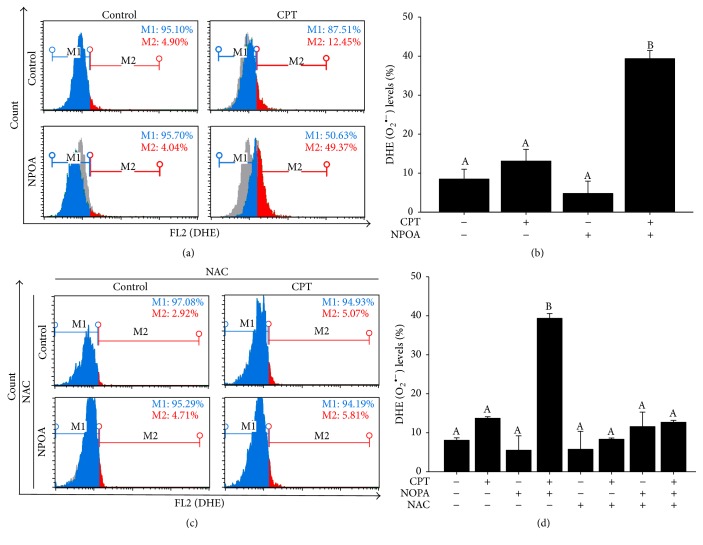
NPOA increased CPT-induced ROS production in H1299 cells. The cells indicate the concentration of CPT and NPOA alone or in combination for 6 h. (a) The levels of ROS production were determined by flow cytometer-based dihydroethidium (DHE) staining assay. (b) The quantification analysis of endogenous ROS. Data are presented as means ± SD. (c) H1299 cells were pretreated with 2 mM NAC for 3 h before CPT alone or CPT and NPOA cotreatment. (d) The quantification analysis (c). Data are presented as means ± SD (^A^ *p* > 0.05, ^B^ *p* < 0.001).

A high level of reactive oxygen species (ROS) is considered to induce apoptosis of cancer cells via mitochondrial pathway [30]. Next, we examined the synergistic effect of NPOA on CPT-induced anti-H1299 cells through upregulating endogenous ROS. The dihydroethidium (DHE) staining can detect endogenous ROS level by combining flow cytometric analyses. We found that the NPOA treatment markedly increased CPT-induced ROS production in H1299 cells compared to the CPT or NPOA treatment alone (Figures 4(a) and 4(b)). These results suggest that NPOA enhanced CPT-induced ROS in H1299 cells may play a pivotal role. On the contrary, the blockage of endogenous ROS by N-acetyl-L-cysteine (NAC), a potent ROS scavenger, moderately reduced endogenous ROS of H1299 cells following CPT and NPOA cotreatment (Figures 4(c) and 4(d)). The result suggests that the CPT and NPOA cotreatment induced apoptosis of H1299 cells through regulating endogenous ROS.

### 3.5. ROS Scavenger Attenuates CPT and NPOA Cotreatment-Induced Apoptosis of H1299 Cells

**Figure 5:**
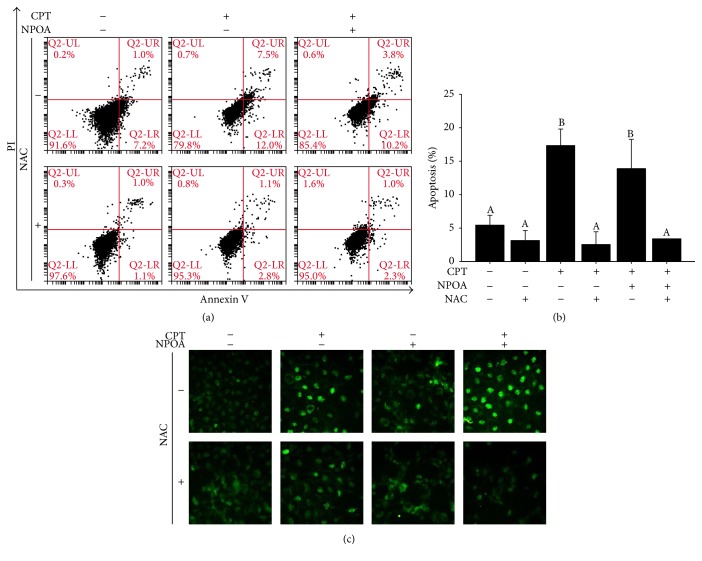
The ROS scavenger attenuated CPT and NPOA cotreatment-induced apoptosis of NSCLC cells. The H1299 cells were pretreated with 2 mM NAC for 3 h and then subject to cotreatment of CPT and NPOA for 24 h, as described in the Materials and Methods section. (a) The cells were double-stained with Annexin V and PI and analyzed by flow cytometer-based detection assay. (b) The quantitative analysis of (a). Data are presented as means ± SD (^A^ *p* > 0.05, ^B^ *p* < 0.001). (c) The results of photography showed the JC-1 green fluorescence image and indicated that NAC rescued the loss of mitochondrial membrane potential of H1299 cells after CPT and NPOA cotreatment. Magnification 200x.

To determine whether the blockage of CPT and NPOA cotreatment-induced ROS production of H1299 cells after treatment with NAC reduces apoptosis, the H1299 cells were precultured with 2 mM NAC for 3 h, followed by CPT and NPOA cotreatment for 6 h. NAC significantly inhibited CPT and NPOA cotreatment-induced apoptosis and depolarization of MMP of H1299 cells (Figure 5). These results suggest that NPOA sensitizes CPT induction towards apoptosis of H1299 cells through modulating endogenous ROS. The results showed that a dramatical loss of MMP (ΔΨ*m*) was induced by CPT and NPOA cotreatment in H1299 cells.

### 3.6. The Upregulation of JNK Phosphorylation and Caspase Activation in H1299 Cells following CPT and NPOA Cotreatment

**Figure 6:**
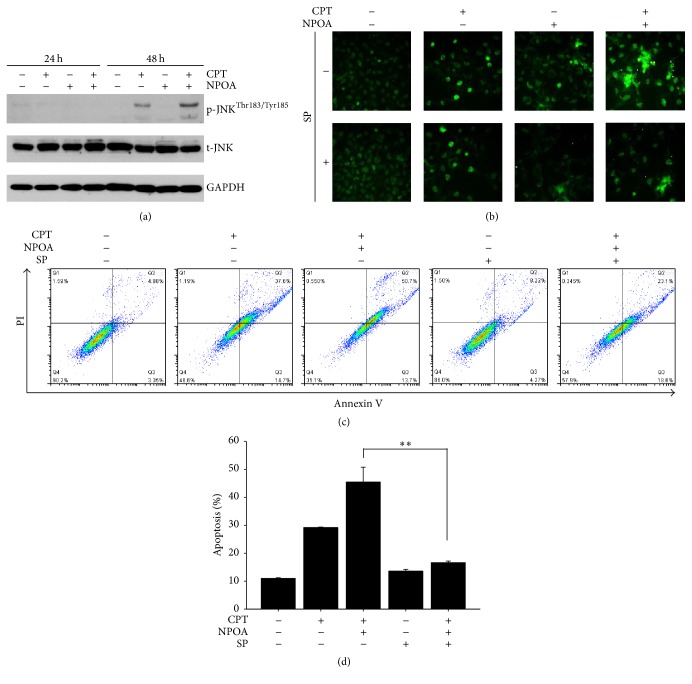
The CPT and NPOA cotreatment induced apoptosis through activating JNK activity. The cells were subject to treatment with vehicle control or the indicated concentration of CPT and NPOA alone or in combination for 24 h and 48 h, respectively. (a) Western blot showed that the CPT and NPOA cotreatment significantly increased levels of JNK phosphorylation (Thr^183^/Tyr^185^) for 48 h. (b) The photograph showed the JC-1 green fluorescence image and indicated that SP600125 (SP) rescued the loss of mitochondrial membrane potential of H1299 cells after CPT and NPOA cotreatment. (c) The effect of SP600125 on CPT and NPOA cotreatment-induced JNK activation in H1299 cells was determined using flow cytometer-based detection assay. The cells were pretreated with JNK inhibitor SP600125 for 3 h and then subject to the treatment of CPT and NPOA alone or their combination. (d) The quantitative analysis of (c). Data are presented as means ± SD (^*∗∗*^ *p* < 0.001). Abbreviations: p-JNK indicates phosphorylation-ERK; t-JNK indicates total-ERK. SP indicates SP600125, a JNK inhibitor. GAPDH was measured as an internal control.

To further explore the mechanism as to how NPOA increases CPT-induced apoptosis of H1299 cells, we investigated whether NPOA increased CPT-induced apoptosis-associated protein in H1299 cells. The requirement of JNK activation for mitochondrial-mediated apoptosis is well documented [31]. Next, we examined whether CPT and NPOA cotreatment induced the JNK phosphorylation of H1299 cells. As described in Figure 6(a), the Western blot analysis showed that CPT and NPOA cotreatment synergistically enhanced the JNK phosphorylation of H1299 cells compared to CPT treatment alone. On the contrary, we used JNK inhibitor, SP600125 (SP), to inhibit JNK activity and to evaluate the effect of JNK inhibition on apoptosis of H1299 cells. The inhibition of JNK activity attenuated CPT and NPOA cotreatment-induced mitochondrial membrane depolarization of H1299 cells (Figure 6(b)). The H1299 cells were precultured with JNK inhibitor (10 *μ*M) for 3 h, followed by cotreatment with CPT and NPOA for 48 h combining the flow cytometry-based analysis. The blockade of JNK attenuated CPT and NPOA cotreatment-induced apoptosis of H1299 cells (Figures 6(c) and 6(d)). Therefore, we suggest that NPOA enhanced CPT-induced mitochondrial-mediated apoptosis of H1299 cells through upregulating JNK activation. These results showed that the NPOA increased CPT-induced apoptosis of H1299 cells through upregulating JNK activation.

## 4. Discussion 

Currently, the treatments for lung cancer include surgical resection, radiation therapy and chemotherapy [32]. The chemotherapeutic treatments, such as platinum-based or CPT-based chemotherapy, have been shown to prolong the survival rate of advanced NSCLC patients. However, the recurrence rate of NSCLC patients after chemotherapy treatment is still up to 50% [33]. Furthermore, the acquired chemoresistant cancer cells are highly correlated with high recurrence and poor prognosis in these patients [34]. Combination chemotherapies, a combination of two or more chemotherapeutic drugs, are widely used to treat cancer, including lung cancer [35]. Unfortunately, the combination chemotherapies do not always enhance the efficacy of anticancer drugs. The combination of docetaxel and cisplatin has been reported to cause antagonism in treating NSCLC EBC-1 (squamous cell carcinoma) and RERF-LCMS cells (adenocarcinoma) [36]. Recently, the antagonism was also observed in 15 human NSCLC cell lines by the treatment of combining gefitinib and cisplatin [37].

In a comparison of combination chemotherapy, the chemosensitizer itself exerts low- or noncytotoxic effects but enhances the efficacy when cotreated with anticancer drugs. For instance, PZ-39, an acetamide-containing compound, was shown to sensitize human breast cancer cell line MCF-7 towards mitoxantrone-induced cell killing [14]. Likewise, amiloride, an acetamide-containing compound, was reported to sensitize four tested human pancreatic cancer cell lines towards erlotinib-induced apoptosis through inhibiting the PI3K/AKT pathway [15]. Accordingly, chemotherapy sensitizer may reduce the risk of side effects and antagonism raised by combination chemotherapies and therefore benefit the cancer treatment.

In this study, we first examined whether an acetamide-containing compound NPOA enhanced the sublethal dose of CPT-induced anti-NSCLC using two NSCLC cells. Our results from trypan blue exclusion assay and Annexin V/PI assay showed that the NPOA sensitized two NSCLC cells towards CPT-induced antiproliferation and apoptosis (Figures 1 and 2). Interestingly, NPOA treatment alone did not exert significant cytotoxicity in two NSCLC cells, whereas a significant increase of Bax in H1299 cells following NPOA treatment was observed. Bax (Bcl-2-associated X protein), a proapoptotic protein of Bcl-2 family, is essential for regulation of apoptosis. For instance, Lalier et al. reported that expression of Bax sensitizes cells to mitochondrial-mediated apoptosis in cancer cells, including lung cancer [38]. We therefore investigated whether the CPT and NPOA cotreatment increased proapoptotic proteins of H1299 cells, including Bax, cleaved caspase 9 and cleaved caspase 3, and mitochondrial depolarization (Figure 3), suggesting that Bax may play a role in contributing to the synergism of CPT and NPOA combination, and the low cytotoxicity of NPOA could be a promising chemosensitizer of CPT-based lung cancer treatment.

Because the synergistic effect of CPT/NPOA on the antiproliferation and apoptosis can be observed in both A549 (*wild type-p53*) and H1299 (*null-p53*) cells (Figure 1), we suggest that the synergistic anti-lung-cancer induced by CPT/NPOA combination may be p53-independent. On the contrary, apoptosis can be induced by two typical signal cascades: the intrinsic (mitochondria-mediated) and the extrinsic (death receptor-mediated) pathways [39]. Fas/Apo-1 (CD95) is mainly involved in the pathway of extrinsic apoptosis; we conducted Western blot assay to determine whether the extrinsic pathway played a role in CPT/NPOA combination-induced apoptosis of lung cancer cells. The results of Western blot assay showed that neither protein level nor cleavage of caspase 8, an apical caspase of extrinsic apoptosis, was affected in both H1299 (Figure 2(c)) and A549 (data not shown). In contrast, CPT/NPOA combination caused the proteolytic activation of caspase-9, an apical caspase of mitochondria-mediated apoptosis in both NSCLC cells (Figure 2(c)). Accordingly, our present results suggest that CPT/NPOA combination-induced apoptosis of lung cancer cells seems to be through mainly mitochondria-mediated pathways. However, the results of our study should not rule out the possibility that other factors might be associated with extrinsic pathways such as caspase 10 being involved in CPT/NPOA-induced apoptosis.

CPT has been shown to inhibit topoisomerase and to induce DNA damage, causing the accumulation of cells at S phase. Therefore, the level of S phase arrest could be an index for the efficacy of CPT treatment [40]. Otherwise, CPT does not majorly arrest cell cycle at S phase, instead at G_1_/S or G_2_/M phases, resulting in attenuation of therapeutic outcome [41]. Our results showed that the accumulation of S phase for vehicle control, CPT alone, NPOA alone, and their combination were 9.03 ± 0.71%, 10.95 ± 0.49%, 11.85 ± 0.92%, and 20.2 ± 1.28%, respectively (Supplementary Figure 1), which were concordant with the activation of *γ*-H_2_AX, a marker of DNA damage. The above-mentioned results suggest that NPOA might synergistically enhance the DNA damage induced by CPT through causing more S phase arrest compared to the treatment of CPT alone.

A previous study found that higher levels of endogenous ROS were detected in many chemoresistant cancer cells [39, 42]. Furthermore, chemoresistant cancer cells have been reported to exert a higher apoptotic threshold by anticancer drugs [43]. Moreover, these chemoresistant cancer cells seem to be more tolerant to increased endogenous ROS by upregulating antioxidant capacity [44]. Additionally, one of the promising strategies for treating chemoresistant cancer cells is through lowering the ROS-induced apoptosis threshold [45]. Therefore, the accumulation of endogenous ROS-generating cells could sensitize cancer cells towards antiproliferation and apoptosis. For example, doxorubicin synergistically enhances CPT-induced apoptosis of cervical carcinoma SiHa cells through upregulating ROS production [46]. It is well known that CPT and its derivatives also increase endogenous ROS of cancer cells, including lung cancer [47] and breast cancer [48]. Besides, a previous study demonstrated that combining topotecan and vorinostat induced apoptosis of two small cell lung cancer (SCLC) cell lines (H209 and H526 cell) through increasing endogenous ROS [49].

Following this, we examined whether NPOA synergistically affected CPT-induced apoptosis of NSCLC cells through modulating ROS production. In this study, we found that NPOA significantly enhanced CPT-induced ROS production from 13.17 ± 1.01% to 39.44 ± 0.09% in H1299 cells (Figure 4). In contrast, our results confirmed that NAC, ROS scavenger, significantly attenuated CPT and NPOA cotreatment-induced ROS production and apoptosis of H1299 cells (Figures 4 and 5), suggesting that the synergistic effect of NPOA on CPT-induced apoptosis of H1299 cells might be through upregulating ROS production.

Endocytosis is a crucial form of active transport in which a cell uptakes molecules such as proteins and anticancer drugs including CPT [50]. Furthermore, previous studies reported that ion-base nanoparticles enhance the oxidative stress and apoptosis by increasing the uptake of ion-based nanoparticles possibly through endocytosis-dependent pathways [51, 52]. For example, zinc oxide nanomaterials enhanced ROS production and apoptosis in two colon cancer cells DLD-1 and SW480 through regulating the pathway of endocytosis [53]. Likewise, a silica nanoparticle of camptothecin was also suggested to enhance apoptosis of colon cancer HT-29 cells via the modulation of endocytosis [54]. Accordingly, we suggest that NPOA might improve the uptake of CPT through endocytosis to sensitize NSCLC H1299 cell towards CPT-induced oxidative stress and apoptosis.

The mitogen-activated protein kinases (MAPK) family is considered to be one of the major ROS-induced signal pathways and is involved in cellular survival, proliferation, and cell death [55]. Among the members of the MAPK family, c-Jun N-terminal protein kinase (JNK) is believed to regulate proapoptotic pathways and lead to cell death [56]. Conversely, the blockade of JNK may cause the resistance of cancer cells towards chemotherapy. For example, Suzuki et al. found that the JNK blockade attenuates two anticancer drugs 5-fluorouracil- (5-FU-) and gemcitabine- (GEM-) induced apoptosis of pancreatic cancer cells [57], suggesting the proapoptotic role of JNK. Therefore, we ought to examine whether the effect of CPT and NPOA cotreatment on apoptosis of NSCLC cells is through upregulating JNK activation (phosphorylation at sites Thr^183^ and Tyr^185^). In our result, JNK was moderately phosphorylated in CPT-treated H1299, whereas CPT and NPOA cotreatment dramatically increased the phosphorylation of JNK in H1299 cells (Figure 6(a)). Otherwise, the blockage of JNK largely decreased CPT and NPOA cotreatment-induced apoptosis (Figure 6(b)), suggesting that the sensitizer role of NPOA in CPT-induced apoptosis of H1299 cells was through modulating the activation of JNK.

## 5. Conclusions

**Figure 7:**
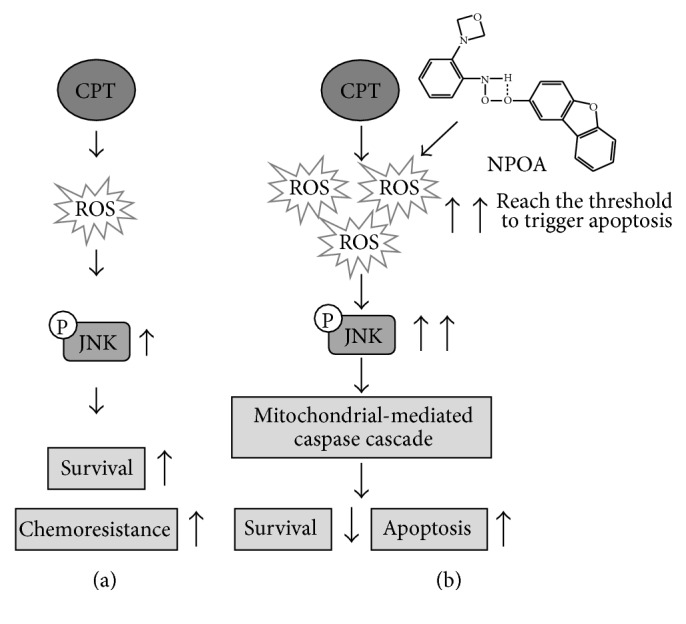
The proposed model shows NPOA sensitized CPT-induced apoptosis of NSCLC cells. (a) With CPT treatment alone, the increased level of ROS does not reach the threshold of apoptosis, whereas CPT-induced JNK activation favors prosurvival, causing an acquired chemoresistance. (b) CPT and NPOA cotreatment dramatically increases the endogenous ROS, JNK activation, and the upregulated expression of prop-apoptotic protein Bax, eventually initiating the mitochondrial-mediated caspase cascade and enhancing the apoptotic population of NSCLC cells.

Taken together, our present results suggest that NPOA sensitizes NSCLC cells towards CPT treatment via upregulating both endogenous ROS and JNK activation, resulting in the increase of S phase accumulation and lowering the threshold of apoptosis (Figure 7). Accordingly, the acetamide derivative NPOA might be a promising sensitizer to CPT-based lung cancer therapy in the future and is worthy of further investigation.

## Supplementary Material


*2.10. Assessment of DNA damage*. After treatments, cells were fixed in 70% ethanol, washed with PBS, and incubated overnight at 4 ℃ in 1 ml of PBS-T with containing 1% BSA and 0.1 μg anti-γH2AX (Ser^139^) monoclonal antibody (Cat. SC-101696, Santa Cruz Biotechnology). Cells were re-suspended and incubated in secondary antibody conjugated with Alexa Fluor 488 (Jackson Laboratory, Bar Harbor, ME, USA) and 1 μg/ml of PI. Stainined cells were analyzed using an Accuri C6 flow cytometer (BD Biosciences).

## References

[1] Siegel R. L., Miller K. D., Jemal A. (2016). Cancer statistics, 2016. *CA: A Cancer Journal for Clinicians*.

[2] Bender E. (2014). Epidemiology: the dominant malignancy. *Nature*.

[3] Manegold C. (2001). Chemotherapy for advanced non-small cell lung cancer: standards. *Lung Cancer*.

[4] Chang A. (2011). Chemotherapy, chemoresistance and the changing treatment landscape for NSCLC. *Lung Cancer*.

[5] Coate L. E., Shepherd F. A. (2011). Maintenance therapy in advanced non-small cell lung cancer: evolution, tolerability and outcomes. *Therapeutic Advances in Medical Oncology*.

[6] Quoix E., Breton J.-L., Gervais R. (2005). A randomised phase II study of the efficacy and safety of intravenous topotecan in combination with either cisplatin or etoposide in patients with untreated extensive disease small-cell lung cancer. *Lung Cancer*.

[7] Fink T. H., Huber R. M., Heigener D. F. (2012). Topotecan/cisplatin compared with cisplatin/etoposide as first-line treatment for patients with extensive disease small-cell lung cancer: final results of a randomized phase III trial. *Journal of Thoracic Oncology*.

[8] Nakashio A., Fujita N., Rokudai S., Sato S., Tsuruo T. (2000). Prevention of phosphatidylinositol 3′-kinase-Akt survival signaling pathway during topotecan-induced apoptosis. *Cancer Research*.

[9] Ramalingam S. S., Foster J., Gooding W., Evans T., Sulecki M., Belani C. P. (2010). Phase 2 study of irinotecan and paclitaxel in patients with recurrent or refractory small cell lung cancer. *Cancer*.

[10] Powell S. F., Beitinjaneh A., Tessema M. (2013). Phase II study of topotecan and bevacizumab in advanced, refractory non-small-cell lung cancer. *Clinical Lung Cancer*.

[11] von Minckwitz G., Costa S. D., Raab G. (2001). Dose-dense doxorubicin, docetaxel, and granulocyte colony-stimulating factor support with or without tamoxifen as preoperative therapy in patients with operable carcinoma of the breast: a randomized, controlled, open phase IIb study. *Journal of Clinical Oncology*.

[12] Tan G.-R., Feng S.-S., Leong D. T. (2014). The reduction of anti-cancer drug antagonism by the spatial protection of drugs with PLA-TPGS nanoparticles. *Biomaterials*.

[13] Yu T., Li S. L., Zhao J. Z., Mason T. J. (2006). Ultrasound: a chemotherapy sensitizer. *Technology in Cancer Research & Treatment*.

[14] Peng H., Dong Z., Qi J. (2009). A novel two mode-acting inhibitor of ABCG2-mediated multidrug transport and resistance in cancer chemotherapy. *PLoS ONE*.

[15] Zheng Y.-T., Yang H.-Y., Li T. (2015). Amiloride sensitizes human pancreatic cancer cells to erlotinib in vitro through inhibition of the PI3K/AKT signaling pathway. *Acta Pharmacologica Sinica*.

[16] Rani P., Pal D., Hegde R. R., Hashim S. R. (2014). Anticancer, anti-inflammatory, and analgesic activities of synthesized 2-(substituted phenoxy) acetamide derivatives. *BioMed Research International*.

[17] Kanagarajan V., Thanusu J., Gopalakrishnan M. (2010). Synthesis and in vitro microbiological evaluation of an array of biolabile 2-morpholino-N-(4,6-diarylpyrimidin-2-yl)acetamides. *European Journal of Medicinal Chemistry*.

[18] Kidwai M., Venkataramanan R., Mohan R., Sapra P. (2002). Cancer chemotherapy and heterocyclic compounds. *Current Medicinal Chemistry*.

[19] Autore G., Caruso A., Marzocco S. (2010). Acetamide derivatives with antioxidant activity and potential anti-inflammatory activity. *Molecules*.

[20] Ayhan-Kilcigil G., Gürkan S., Çoban T., Özdamar E. D., Can-Eke B. (2012). Synthesis and evaluation of antioxidant properties of novel 2-[2-(4-chlorophenyl) benzimidazole-1-yl]-N-(2-arylmethylene amino) acetamides and 2-[2-(4-chlorophenyl) benzimidazole-1-yl]-N-(4-oxo-2-aryl-thiazolidine-3-yl) acetamides-I. *Chemical Biology and Drug Design*.

[21] Chan H.-C., Kuo S.-C., Huang L.-J., Liu C.-H., Hsu S.-L. (2003). A phenylacetate derivative, SCK6, inhibits cell proliferation via G1 cell cycle arrest and apoptosis. *European Journal of Pharmacology*.

[22] Hanif F., Perveen K., Jawed H. (2014). N-(2-hydroxyphenyl)acetamide (NA-2) and Temozolomide synergistically induce apoptosis in human glioblastoma cell line U87. *Cancer Cell International*.

[23] van Kuppeveld F. J. M., Johansson K.-E., Galama J. M. D. (1994). Detection of mycoplasma contamination in cell cultures by a mycoplasma group-specific PCR. *Applied and Environmental Microbiology*.

[24] Chen W.-C., Wang S.-Y., Chiu C.-C. (2013). Lucidone suppresses hepatitis C virus replication by Nrf2-mediated heme oxygenase-1 induction. *Antimicrobial Agents and Chemotherapy*.

[25] Chou T. C., Talalay P. (1984). Quantitative analysis of dose-effect relationships: the combined effects of multiple drugs or enzyme inhibitors. *Advances in Enzyme Regulation*.

[26] Chou T. C., Talalay P. (1981). Generalized equations for the analysis of inhibitions of Michaelis-Menten and higher-order kinetic systems with two or more mutually exclusive and nonexclusive inhibitors. *European Journal of Biochemistry*.

[27] Hsu Y.-Y., Chen C.-S., Wu S.-N., Jong Y.-J., Lo Y.-C. (2012). Berberine activates Nrf2 nuclear translocation and protects against oxidative damage via a phosphatidylinositol 3-kinase/Akt-dependent mechanism in NSC34 motor neuron-like cells. *European Journal of Pharmaceutical Sciences*.

[28] Chiu C.-C., Chen J. Y.-F., Lin K.-L. (2010). P38 MAPK and NF-*κ*B pathways are involved in naphtho[1,2-b] furan-4,5-dione induced anti-proliferation and apoptosis of human hepatoma cells. *Cancer Letters*.

[29] Perelman A., Wachtel C., Cohen M., Haupt S., Shapiro H., Tzur A. (2012). JC-1: alternative excitation wavelengths facilitate mitochondrial membrane potential cytometry. *Cell Death and Disease*.

[30] Sen N., Das B. B., Ganguly A., Mukherjee T., Bandyopadhyay S., Majumder H. K. (2004). Camptothecin-induced imbalance in intracellular cation homeostasis regulates programmed cell death in unicellular hemoflagellate *Leishmania donovani*. *The Journal of Biological Chemistry*.

[31] Dhanasekaran D. N., Reddy E. P. (2008). JNK signaling in apoptosis. *Oncogene*.

[32] Reck M., Heigener D. F., Mok T., Soria J.-C., Rabe K. F. (2013). Management of non-small-cell lung cancer: recent developments. *The Lancet*.

[33] Uramoto H., Tanaka F. (2014). Recurrence after surgery in patients with NSCLC. *Translational Lung Cancer Research*.

[34] Gallego M.-A., Ballot C., Kluza J. (2008). Overcoming chemoresistance of non-small cell lung carcinoma through restoration of an AIF-dependent apoptotic pathway. *Oncogene*.

[35] DeVita V. T., Chu E. (2008). A history of cancer chemotherapy. *Cancer Research*.

[36] Wang H. (2000). Combined effect of docetaxel and cisplatin for non-small cell lung cancer cell lines in vitro. *Nagoya Journal of Medical Science*.

[37] Tsai C.-M., Chen J.-T., Stewart D. J. (2011). Antagonism between gefitinib and cisplatin in non-small cell lung cancer cells: why randomized trials failed?. *Journal of Thoracic Oncology*.

[38] Lalier L., Cartron P.-F., Juin P. (2007). Bax activation and mitochondrial insertion during apoptosis. *Apoptosis*.

[39] Mor G., Montagna M. K., Alvero A. B. (2008). Modulation of apoptosis to reverse chemoresistance. *Methods in Molecular Biology*.

[40] Seiler J. A., Conti C., Syed A., Aladjem M. I., Pommier Y. (2007). The intra-S-phase checkpoint affects both DNA replication initiation and elongation: single-cell and -DNA fiber analyses. *Molecular and Cellular Biology*.

[41] Goldwasser F., Shimizu T., Jackman J. (1996). Correlations between S and G2 arrest and the cytotoxicity of camptothecin in human colon carcinoma cells. *Cancer Research*.

[42] Fraser M., Leung B., Jahani-Asl A., Yan X., Thompson W. E., Tsang B. K. (2003). Chemoresistance in human ovarian cancer: the role of apoptotic regulators. *Reproductive Biology and Endocrinology*.

[43] Wang C.-Y., Cusack J. C., Liu R., Baldwin A. S. (1999). Control of inducible chemoresistance: enhanced anti-tumor therapy through increased apoptosis by inhibition of NF-*κ*B. *Nature Medicine*.

[44] Trachootham D., Alexandre J., Huang P. (2009). Targeting cancer cells by ROS-mediated mechanisms: a radical therapeutic approach?. *Nature Reviews Drug Discovery*.

[45] Trachootham D., Lu W., Ogasawara M. A., Valle N. R.-D., Huang P. (2008). Redox regulation of cell survival. *Antioxidants and Redox Signaling*.

[46] Ha S. W., Kim Y. J., Kim W., Lee C. S. (2009). Antitumor effects of camptothecin combined with conventional anticancer drugs on the cervical and uterine squamous cell carcinoma cell line SiHa. *Korean Journal of Physiology and Pharmacology*.

[47] Brea-Calvo G., Siendones E., Sánchez-Alcázar J. A., de Cabo R., Navas P. (2009). Cell survival from chemotherapy depends on NF-*κ*B transcriptional up-regulation of coenzyme Q biosynthesis. *PLoS ONE*.

[48] Timur M., Akbas S. H., Ozben T. (2005). The effect of Topotecan on oxidative stress in MCF-7 human breast cancer cell line. *Acta Biochimica Polonica*.

[49] Bruzzese F., Rocco M., Castelli S., Di Gennaro E., Desideri A., Budillon A. (2009). Synergistic antitumor effect between vorinostat and topotecan in small cell lung cancer cells is mediated by generation of reactive oxygen species and DNA damage-induced apoptosis. *Molecular Cancer Therapeutics*.

[50] Liu Y.-S., Cheng R.-Y., Lo Y.-L. (2016). Distinct CPT-induced deaths in lung cancer cells caused by clathrin-mediated internalization of CP micelles. *Nanoscale*.

[51] Nel A., Xia T., Mädler L., Li N. (2006). Toxic potential of materials at the nanolevel. *Science*.

[52] Schins R. P. F. (2002). Mechanisms of genotoxicity of particles and fibers. *Inhalation Toxicology*.

[53] Setyawati M. I., Tay C. Y., Leong D. T. (2013). Effect of zinc oxide nanomaterials-induced oxidative stress on the p53 pathway. *Biomaterials*.

[54] Botella P., Abasolo I., Fernández Y. (2011). Surface-modified silica nanoparticles for tumor-targeted delivery of camptothecin and its biological evaluation. *Journal of Controlled Release*.

[55] Park W. H. (2013). The effect of MAPK inhibitors and ROS modulators on cell growth and death of H_2_O_2_-treated HeLa cells. *Molecular Medicine Reports*.

[56] Wang H.-B., Ma X.-Q. (2014). Activation of JNK/p38 pathway is responsible for *α*-methyl-n-butylshikonin induced mitochondria-dependent apoptosis in SW620 human colorectal cancer cells. *Asian Pacific Journal of Cancer Prevention*.

[57] Suzuki S., Okada M., Shibuya K. (2015). JNK suppression of chemotherapeutic agents-induced ROS confers chemoresistance on pancreatic cancer stem cells. *Oncotarget*.

