# Supplementary material for: An Acetamide Derivative as a Camptothecin Sensitizer for Human Non-Small-Cell Lung Cancer Cells through Increased Oxidative Stress and JNK Activation
Source: Oxid Med Cell Longev. 2016 Oct 24;2016:9128102. doi: 10.1155/2016/9128102 (PMC5098095; doi:10.1155/2016/9128102)
Supplement: Supplementary file 1 — 2.10. Assessment of DNA damage. After treatments, cells were fixed in 70% ethanol, washed with PBS, and incubated overnight at 4 ℃ in 1 ml of PBS-T with containing 1% BSA and 0.1 μg anti-γH2AX (Ser139) monoclonal antibody (Cat. SC-101696, Santa Cruz Biotechnology). Cells were re-suspended and incubated in secondary antibody conjugated with Alexa Fluor 488 (Jackson Laboratory, Bar Harbor, ME, USA) and 1 μg/ml of PI. Stainined cells were analyzed using an Accuri C6 flow cytometer (BD Biosciences). [file 9128102.f1.pdf]

Supplementary Table 1

(a)

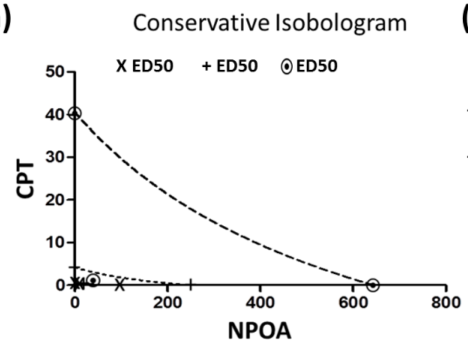

(b)

Combination	Mean ± SD at			
Compound	ED50	ED75	ED90	Influence
NPOA	0.655 ± 0.0542	0.195 ± 0.017	0.086 ± 0.0121	Synergistic

Supplementary Table l: The synergistic effects of CPT and NPOA combination on anti-proliferation of NSCLC cells. H1299 cells were treated with combinations of indicated concentrations of CPT and NPOA combination for 48 h respectively. The survival rate of H1299 cells was measured using Trypan blue exclusion assay. (a) The synergistic effects of CPT and NPOA combination are evaluated using the program CalcuSyn and the lines represent the effective doses (EDs) of the effects of the two compounds to achieve 50, 75, or 90% inhibition. The dots represent the experimental doses used for achieving the above inhibitory effects. (b) The table represents the mean values of CI, which represent the degree of CPT and NPOA interactions, < 1 for synergistic-, = 1 for additive-, and > 1 for antagonistic effects respectively. The results are means±S.D. from at least three independent experiments.
