# Supplementary material for: An Acetamide Derivative as a Camptothecin Sensitizer for Human Non-Small-Cell Lung Cancer Cells through Increased Oxidative Stress and JNK Activation
Source: Oxid Med Cell Longev. 2016 Oct 24;2016:9128102. doi: 10.1155/2016/9128102 (PMC5098095; doi:10.1155/2016/9128102)
Supplement: Supplementary file 2 [file 9128102.f2.pdf]

Supplementary Figure 1

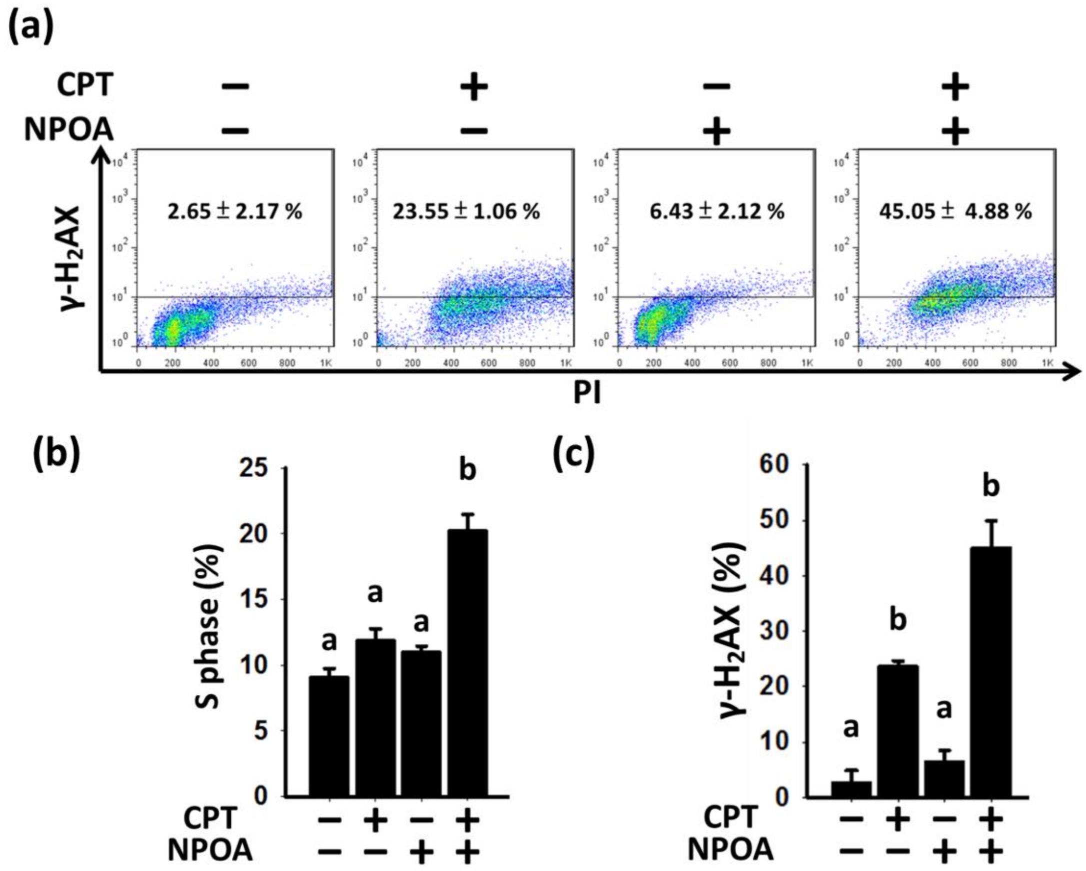

Supplementary Figure l: The effect of CPT/NPOA combination on cell cycle distribution and the induction of DNA damage in NSCLC cells. (a) H1299 cells after growth for 24 h in CPT and NPOA treatment alone or a combination. (b) The quantitative analysis of cell cycle S phase accumulation of (a). (c) The quantitative analysis of γ-H_2AX activation of (a) ($p^a > 0.05$, $p^b < 0.001$).
